# rSalvador: An R Package for the Fluctuation Experiment

**DOI:** 10.1534/g3.117.300120

**Published:** 2017-10-30

**Authors:** Qi Zheng

**Affiliations:** Department of Epidemiology and Biostatistics, Texas A&M School of Public Health, College Station, Texas 77843

**Keywords:** Luria-Delbrück protocol, practical nonidentifiability, likelihood ratio test

## Abstract

The past few years have seen a surge of novel applications of the Luria-Delbrück fluctuation assay protocol in bacterial research. Appropriate analysis of fluctuation assay data often requires computational methods that are unavailable in the popular web tool FALCOR. This paper introduces an R package named rSalvador to bring improvements to the field. The paper focuses on rSalvador’s capabilities to alleviate three kinds of problems found in recent investigations: (i) resorting to partial plating without properly accounting for the effects of partial plating; (ii) conducting attendant fitness assays without incorporating mutants’ relative fitness in subsequent data analysis; and (iii) comparing mutation rates using methods that are in general inapplicable to fluctuation assay data. In addition, the paper touches on rSalvador’s capabilities to estimate sample size and the difficulties related to parameter nonidentifiability.

Nearly 75 yr ago, Luria and Delbrück (1943) proposed an experimental protocol that today is known variously as the fluctuation test, the fluctuation experiment, and the fluctuation assay. This innovative experimental protocol is so far the preferred method for determining microbial mutation rates in the laboratory. Because computational methods for inferring mutation rates from fluctuation assay data seemed complicated to most biologists, for over half a century the fluctuation experiment had been an investigative tool only for mathematically minded biologists or biologists who could find adequate computational assistance. The advent of SALVADOR (Zheng 2002), a package written in the Wolfram language (Wolfram Research, Inc. 2016), induced a surge of applications of the fluctuation experiment by investigators who otherwise would not have considered the classical protocol. However, because SALVADOR runs in the proprietary Mathematica (Wolfram Research, Inc. 2016) environment, it raised a new, albeit lesser, barrier to the widespread use of the fluctuation experiment. It was Hall *et al.* (2009) who helped bench scientists break free from the shackles of proprietary software. Their web tool, FALCOR, enables investigators to perform basic analysis of fluctuation assay data in a way no more arduous than calculating the sample mean and sample SD. Numerous researchers, empowered by this convenient web tool, used the fluctuation experiment in tandem with DNA sequencing techniques in their research. As a result, an astonishing flood of novel applications of the fluctuation experiment followed. FALCOR was an effective catalyst for a marriage between the classical fluctuation experiment and modern DNA sequencing techniques.

With FALCOR’s popularity came a disturbing irony. As noted in a recent review (Zheng 2015b), FALCOR offers only methods that were reviewed in 2000 (Rosche and Foster 2000). Many applications require methods that either were developed after 2000 or are to be developed. Researchers who acquainted themselves with the analysis of fluctuation assay data via FALCOR were often under the impression that all fluctuation assay data could be analyzed by a standardized approach, which Hall *et al.* (2009) advocated. Owing to this mistaken perception, investigators in a large fraction of recent studies adopted a common method to analyze their fluctuation assay data, paying little attention to important features that distinguished their experiments. A conscientious practitioner would then be baffled when Ycart (2013) sounded a note of warning, in stark contrast to the sanguine views of Hall *et al.* (2009). The rhetorical question “Can estimates be trusted?”, sparked by a common mathematical assumption about cell life spans, seemed to obliterate bench scientists’ hopes for a practical way to analyze fluctuation assay data on their own. Moreover, Hamon and Ycart (2012) voiced their complaints about the extensive numerical problems that were supposedly caused by the maximum likelihood method. Besides clarifying the disquieting confusion prompted by these issues, the present article uses rSalvador 1.7 (Zheng 2017a) to guide readers in the analysis of their fluctuation assay data.

rSalvador was first released in April 2014. It was written as an R package, but the most compute-intensive parts, as in SALVADOR, were coded in the C programming language. rSalvador is not merely an R adaptation of SALVADOR, for it affords new methods developed after the last release of SALVADOR. This article describes rSalvador’s capabilities by focusing on three kinds of inadequacies commonly found in recent analyses of fluctuation assay data. The three kinds of inadequacies are: (i) resorting to partial plating without properly accounting for the effects of partial plating; (ii) conducting attendant fitness assays without accounting for mutants’ relative fitness when calculating estimates of mutation rates; and (iii) comparing mutation rates using methods that are in general inapplicable to fluctuation assay data.

## Relevant Assumptions

The fluctuation experiment is conceptually simple. Although neither mutations nor mutants are directly observable, a mutant can be made visible by allowing it to form a colony on a solid culture. As a result, analysis of fluctuation assay data revolves around inferring the number of mutations from the number of mutants in a test tube. This challenging task relies critically on mathematical models that bridge the gap between mutations and mutants. Like mathematical models for any other purposes, a mathematical model for fluctuation assay data depends on simplifying assumptions. Acquiring a sound understanding of the key assumptions not only helps experimentalists to better design experiments, but also enables them to analyze their data more confidently. Major assumptions underlying common mathematical models for fluctuation assay data include the following.

A1. Cells (nonmutants and mutant alike) undergo unimpeded growth in a liquid culture (contained in a test tube). In other words, cells are in logarithmic phase (also known as exponential phase).
A2. When a cell divides, the probability is a constant *p* that one of the two daughter cells is a mutant. The probability is 1 − *p* that both daughter cells are nonmutants. Therefore, there is no possibility of a nonmutant splitting into two daughter mutants.
A3. Mutants grow at the same rate as nonmutants. As a consequence, the relative fitness is unity.
A4. Back mutation is negligible, and hence is not considered.
A5. Cell death is negligible, and hence is not considered. As Kendal and Frost (1988) rightly asserted, this assumption is not a problem when dealing with bacterial cells (in contrast to somatic cells). But there is an important exception. When the Luria-Delbrück protocol is used to study antibiotic-induced mutations, as in the study of Cairns *et al.* (2017), cells must grow in the presence of an antibiotic. The dose administered should be sufficiently low so as not to cause considerable death of nonmutants. Otherwise, owing to death of large numbers of nonmutants, unimpeded growth of antibiotic-resistant mutants will grossly inflate the mutant frequency. This caution should not be confused with the use of a lethal dose of an antibiotic after the cells are plated, as a selective agent.
A6. Every mutant exiting prior to plating is capable of growing into a colony after the plating process. In other words, the plating efficiency is perfect.
A7. Cell life span obeys the negative exponential distribution.
A8. The total numbers of nonmutants immediately before plating, denoted by *N_t_*, are approximately the same across tubes within an experiment.
A9. Nonmutants die immediately after plating. In other words, selection after plating is so lethal that postplating mutation is negligible. Note that postplating mutation was of interest to Lang and Murray (2008), and to Ford *et al.* (2013).
A10. When a nonmutant undergoes a mutation, the resulting daughter mutant is capable of immediately manifesting the mutation’s phenotype. This assumption thus excludes the possibility of phenotypic delay, a concept that can be traced to Newcombe (1948).
A11. *N*_0_, the number of cells used to seed a test tube, is small enough to guarantee that the *N*_0_ seeding cells contain no mutants.

A useful mutation model yields a so-called mutant distribution, which describes the probability distribution of the number of mutants, and which usually involves a parameter customarily denoted by *m*, the expected number of mutations per tube. Once an accurate estimate $\hat{m}$ of *m* is inferred from fluctuation assay data using an appropriate mutant distribution, the mutation rate is determined by

$$p=\frac{\hat{m}}{N_{t}-N_{0}}\approx \frac{\hat{m}}{N_{t}}.$$ (1)

For a detailed discussion of the mutation rate *p*, the reader is referred to Zheng (2017c).

Almost by definition, a simplifying assumption is a deviation from biological reality. One is doomed to disappointment when checking any of the above assumptions outside the context of what the fluctuation experiment aims to achieve. As the point of the fluctuation experiment is to estimate the fundamental parameter *m*, the validity of the assumptions (A1)–(A11) should be assessed by whether they allow acceptable estimates of *m* to be extracted from fluctuation assay data. Although limited research has been conducted in this regard, the assumptions seem to be less worrisome than originally thought. For example, the assumption (A7), the most contrived at first glance, was recently found to be an acceptable assumption.

A peculiar feature of all mutant distributions is a lack of explicit analytic expressions. A mutant distribution must therefore be identified by its probability generating function (PGF). If *Y* denotes the random number of mutants, the PGF of *Y* is the function G(∙) defined by $G\left(z\right)=E\left[z^{Y}\right]\;$. rSalvador encompasses five mutation distributions. The first mutant distribution is due to Lea and Coulson (1949). This distribution depends on all the above 11 assumptions, and its PGF is

$$G_{1}\left(z;m,\emptyset \right)=exp\left[\frac{m}{\text{∅}}\left(\frac{1}{z}-1\right)\log\left(1-\emptyset z\right)\right].$$ (2)

Here, $\emptyset =1-N_{0}/N_{t}$ is strictly less than unity (Zheng 2002). But φ is assumed to be unity by convention. Strictly speaking, the Lea-Coulson mutant distribution refers to the special case where the PGF is of the form

$$G_{1}^{*}\left(z;m\right)=exp\left[m\left(\frac{1}{z}-1\right)\log\left(1-z\right)\right].$$ (3)

Nádas *et al.* (1996) were the first to suspect that setting ∅ = 1 could have adverse effects on the estimation of *m*. However, in practice, ∅ > 0.999 almost always holds true. Under such a condition, the effects of ∅ are negligible. rSalvador is the only software tool that permits the user to specify a value smaller than unity for ∅, mainly for educational purposes.

The second mutant distribution resulted from an attempt to relax the assumption (A3) by Mandelbrot (1974). Because Koch (1982) made a similar attempt to allow for differential growth rates between nonmutants and mutants, the mutant distribution is usually called the Mandelbrot-Koch distribution. The following PGF is a generalization of the PGF $G_{1}^{*}\left(z;m\right):$

$$G_{2}\left(z;m,w\right)=exp\left(-m+\frac{m}{w}\sum_{k=1}^{\infty }B\left(k,\;1+w^{-1}\right)z^{k}\right)$$ (4) .

Here *B* denotes the usual beta function. The parameter *w* is relative fitness, that is, the ratio of the growth rate of mutants to that of nonmutants.

The third mutant distribution can be traced to Armitage (1952), who was the first to attempt to relax the assumption (A6). The resulting mutant distribution was further studied by Stewart *et al.* (1990). The PGF of that distribution is

$$G_{3}\left(z;m,\epsilon \right)=exp\left(m\xi \frac{(1-z)\log\left[\epsilon (1-z)\right]}{1+\;\xi z}\right).$$ (5)

Here, ξ = ϵ / (1− ϵ). The parameter ϵ is called the plating efficiency, which denotes the portion of culture plated or the probability that a mutant forms a visible colony after the plating process.

The fourth mutant distribution is a special case of the relatively new $B^{0}$ distribution (Zheng 2010). The $B^{0}$ distribution has two parameters, *A* and *k*, and is denoted by $B^{0}\left(A,k\right).$ The PGF of a $B^{0}\left(A,k\right)$ variable is

$$G_{4}\left(z;A,k\right)={\left(\frac{1}{1-A\left(z^{-1}-1\right)\log\left(1-z\right)}\right)}^{k}.$$ (6)

The $B^{0}$ distribution’s usefulness lies in its ability to relax the assumption (A8).

The last mutant distribution derives from Haldane’s mutation model (Zheng 2005). Because cell growth is synchronous in the Haldane model, cell life spans are nonrandom. This is in violation of the assumption (A7). The Haldane mutant distribution is indexed by the mutation rate *p* and the number of elapsed cell generations *g*. rSalvador includes the Haldane model mainly for educational purposes, and it relies on algorithms developed by Zheng (2007).

## Classical Analysis

In most published applications of the fluctuation experiment, inference about *m* relied on the Lea-Coulson mutant distribution (Lea and Coulson 1949) defined by the PGF $G_{1}^{*}\left(z;m\right)$ given in Equation (3). The Lea-Coulson distribution owes its popularity partly to the work by Ma *et al.* (1992). SALVADOR 1.0 (Zheng 2002) was the first publicly available software package allowing the user to calculate maximum likelihood (ML) estimates of *m* under the Lea-Coulson model. The web tool FALCOR (Hall *et al.* 2009) rendered this capability more easily accessible to bench scientists. But FALCOR does not automate the process of computing confidence intervals (C.I.s) for *m*. As a result, the user is forced to compute C.I.s outside FALCOR by following instructions given on FALCOR’s website.

rSalvador has a unique advantage. Since SALVADOR 2.0 (Zheng 2005), the expected Fisher information has been replaced by the observed Fisher information in all algorithms for computing point and interval estimates of *m*. As a consequence, all C.I.s produced by SALVADOR (or rSalvador) are based on the likelihood ratio method. Efron and Hinkley (1978) were among the first to observe that likelihood ratio C.I.s are preferable to the commonly used Wald-type C.I.s. Raue *et al.* (2009) referred to likelihood ratio C.I.s as finite sample C.I.s, to underscore the superiority of likelihood ratio C.I.s over Wald-type C.I.s. rSalvador (including SALVADOR) is so far the only fluctuation assay tool that advocates likelihood ratio C.I.s. One distinctive feature of the likelihood ratio C.I. for *m* is that it is usually asymmetric about the ML estimate of *m*. As an example, consider the well-known Demerec data (Demerec 1945). The ML point estimate of *m* is

> newton.LD(demerec.data)

[1] 10.84383

But a 95% likelihood ratio C.I. for *m* is

> confint.LD(demerec.data)

[1] 8.650538 13.194765

The center of this C.I. is at 10.92, not 10.84—the ML estimate of *m*. Unlike the Wald-type C.I.s, a likelihood ratio C.I. requires iterative computing. The user can view the iterative process by setting the option show.iter = T in the above command.

As an educational feature, rSalvador allows the user to specify an arbitrary value between 0 and 1 for the parameter φ given in Equation (2). In the Demerec experiment, *N*_0_ = 90 and *N_t_* = 1.9 × 10^8^ (Zheng 2002). However, if the experiment were terminated prematurely at *N_t_* = 900, one would have φ = 1 − 90/900 = 0.9. A noticeably different estimate of *m* would result, as predicted by Nádas *et al.* (1996).

> newton.LD(demerec.data,phi = 0.9)

[1] 18.92394

The simple Lea-Coulson model relies on each of the assumptions (A1)–(A11), which may belie the model’s practical usefulness. One source of skepticism is the assumption (A7), as cell life spans do not obey the negative exponential distribution. It was this assumption that triggered the rhetorical question “Can estimates be trusted?” In a study of the Haldane model, Zheng (2007) compared the Lea-Coulson model with the Haldane model and found that the two models produced comparable estimates of mutation rates. This finding suggests that the assumption (A7) is acceptable, because the two models assume drastically different cell life distributions. A simulation study by Ycart (2013) reached essentially the same conclusion. Still, Ycart (2013) suggested using a cell life distribution derived from a particular yeast study as a universal cell life distribution. In response to Ycart’s suggestion, Gillet-Markowska *et al.* (2015) emphasize that “there is no universal cellular division time model as it depends on experimental conditions like the strain or the media.”

## Accounting for Relative Fitness

Classical analysis of fluctuation assay data excludes the possibility of differential growth rates between mutants and nonmutants. Recognition of the importance of allowing for this possibility can be traced to Lieb (1951), who highlighted the *P*_0_ method of Luria and Delbrück (1943) for its capability to accommodate differential growth rates. Using the mutant distribution defined by the PGF in Equation (4), Zheng (2002) developed algorithms for computing ML estimates of *m* and *w*. These algorithms rely on the expected Fisher information that must be computed by truncating infinite series. Because computing infinite series could be prohibitively expensive in practice, Zheng (2005) improved these algorithms by replacing the expected Fisher information with the observed Fisher information, which not only obviated the problem of infinite series but also made possible the computation of likelihood ratio C.I.s. A comparison between Equations (32) and (46) in Zheng (2005) clarifies the distinction between the expected and the observed Fisher information. The elegant work of Efron and Hinkley (1978) provides helpful information on this topic.

However, Hamon and Ycart (2012) drew renewed attention to the Mandelbrot-Koch model by making the following observation.

Indeed, the likelihood and its derivatives can be computed by iterative algorithms: theoretically at least, the problem could be considered solved. This is not so in practice, mainly because the multiple sums that must be computed by the optimization algorithm make it quite unstable. According to the numerous tests that we have made, the ML estimates cannot be reliably computed for samples whose maximum exceeds 1000.

Such a disastrous outcome is unreproducible, as it was an artifact of the computer code of Hamon and Ycart. The improved algorithms for computing ML estimates given by Zheng (2005) had been thoroughly tested using simulated fluctuation experiments in which the maximum mutant counts far exceeded 5000. A diagnostic clue to the enigma is on p. 1258 of Hamon and Ycart (2012) below an expression for the expected Fisher information, denoted by *I* (α, ρ).

Fortunately the partial sums increase, so that when computing the inverse *I^−1^*(α, ρ) the sum of the first *m* terms yields conservative confidence intervals; yet we do not consider it satisfactory.

Unaware of the advantages of using the observed Fisher information, Hamon and Ycart (2012) still used algorithms reliant on the expected Fisher information to compute ML estimates. This then-outdated practice led to an exaggerated sense of disaster, so Hamon and Ycart recommended the generating function (GF) method as a remedy. They envisioned future experiments in which cultures would contain enormous numbers of mutants to allow the experimentalist to harness the GF method’s potential. This would imply the abandonment of the Luria-Delbrück protocol, as large numbers of mutants must be directly counted, without the aid of plating. Such views caused confusion. For example, a simulation study of the GF method led Gillet-Markowska *et al.* (2015) to emphasize the importance of avoiding exceedingly large mutant counts.

[T]he precision on the estimation of *m* (and by consequence the estimation of *μ*) is higher for the smallest values of *m*. Therefore, users should not outgrow the cultures in order to limit the number of mutants that grow on selective plates.

**Figure 1:**
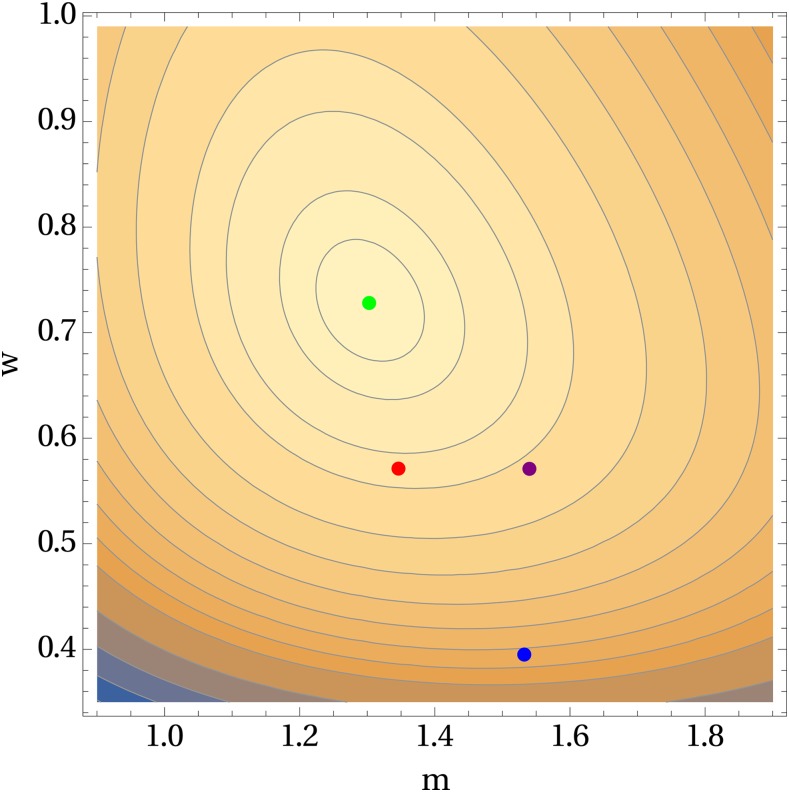
Contours of the log-likelihood function for the data given in Table 3 of Rosche and Foster (2000). The four colored dots represent estimates of *m* and *w*. The green dot indicates the ML estimates, the blue dot indicates the GF estimates via *flan*, the purple dot indicates the GF estimates via *bz-rates*, and the red dot indicates the GF estimates by an earlier implementation of the GF method.

The exploration of the GF method by Hamon and Ycart (2012) offers valuable lessons, which I here illustrate using the experimental data given in Table 3 of Rosche and Foster (2000). Eight of the 60 mutant counts were excluded in the analysis by Hamon and Ycart (2012), but I shall use the intact data. One reason for choosing this data set is that it contains the largest mutant count ever reported—3000. At least three versions of the GF method are available: that given by Hamon and Ycart (2012) via the R code attendant to their paper, that given by the web tool *bz-rates* (Gillet-Markowska *et al.* 2015), and that given by the R package flan (Mazoyer *et al.* 2017). ML estimates of *m* and *w*, along with estimates produced by the three versions of the GF method, are displayed in Figure 1. Judging by the contours of the log-likelihood function, the ML method outperforms all three versions of the GF method. The large mutant count did not derail the ML method, contradicting the observation of Hamon and Ycart (2012).

> newton.joint.MK(cairns.foster.data)

[1] 1.3027909 0.7281044

Likelihood ratio C.I.s for *m* and *w* can be computed separately as follows.

> confint.profile.m(cairns.foster.data)

[1] 0.9855115 1.6749828

> confint.profile.w(cairns.foster.data)

[1] 0.5209636 1.0298620

The exploration of the GF method drew attention to the issue of parameter identifiability. Only recently did investigators in systems biology and related fields recognize practical identifiability as a problem distinct from the better-known problem of structural identifiability (Raue *et al.* 2009). Practical identifiability considers the effects of the amount and quality of data. Computational difficulties encountered with the Mandelbrot-Koch mutant distribution may also be viewed as practical nonidentifiability. The inability to estimate *w* in the examples given by Mazoyer *et al.* (2017) is indicative of practical nonidentifiability. In addition, the C.I.s that extend to zero in these examples are a symptom of the same problem. No multi-parameter mutant distribution seems to be immune to practical nonidentifiability. Angerer (2001) was perhaps the first to document computational difficulties that were due to practical nonidentifiability. In my own simulation exploration of the mutant distribution used by Lang and Murray (2008) and by Ford *et al.* (2013), I also found practical nonidentifiability a stubborn problem.

A motivation for studying the GF method was to improve the estimation of the fitness parameter *w*. Simulation suggests that it is uneconomical (in terms of sample size) to use the Luria-Delbrück protocol as a tool for measuring bacterial fitness. The traditional fitness assay (also known as the competition assay) is a more efficient tool, which has been increasingly conducted in tandem with fluctuation assays in recent studies (Scanlan *et al.* 2015). The Mandelbrot-Koch mutant distribution enables the investigator to incorporate information on *w* in the analysis of fluctuation assay data. For instance, if a fitness assay yields an estimated *w* of 0.73 in the above example, one obtains an estimate of *m* by

> newton.MK(cairns.foster.data,w = 0.73)

[1] 1.30215

In the absence of attendant fitness assays, the investigator may use joint ML estimation cautiously as a tool to obtain an estimate of *m* adjusted for the nuisance parameter *w*. In doing so, the issue of parameter identifiability should be kept in mind. While the ML approach is unsuitable for unrealistically large mutant counts, it remains the method of choice for real-world applications. An experimentalist is rarely able to count 500 mutant colonies per culture (Boe *et al.* 1994), let alone 5000 mutant colonies per culture. The experimentalist resorts to partial plating if a poorly designed experiment yields unwieldy mutant counts.

## Accounting for Plating Efficiency

**Figure 2:**
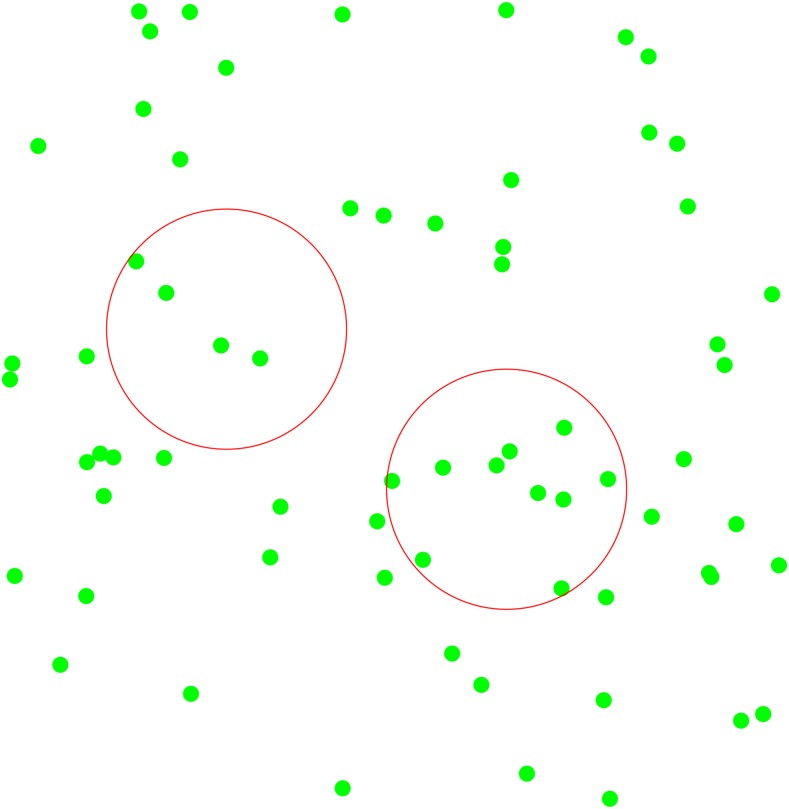
This diagram draws an analogy between a three-dimensional liquid culture and a slice of raisin bread, to help explain the randomness induced by partial plating. The green points, each symbolizing a mutant, are randomly dispersed in the square area. The two circles indicate two possible ways of sampling (plating) an equal portion of the square area that represents a three-dimensional liquid culture.

When an experimentalist plates only a portion of each culture, we refer to the actually plated proportion as the experiment’s plating efficiency, often denoted by ϵ. A culture before plating is densely populated by cells, but mutants constitute only a minute fraction of the cell population. As a result, mutants are sparsely and randomly dispersed in the culture. If 10% (*i.e.*, ϵ=0.1) of a culture is plated, in general, the experimentalist does not transfer exactly 10% of the mutants onto a selective culture—the actual number of mutants transferred is a random number. Although a liquid culture resides in a three-dimensional tube, the randomness induced by partial plating is more easily understood by analogy with a slice of raisin bread, as illustrated by Figure 2.

Partial plating is common, as it is a practical measure to circumvent overwhelmingly large mutant counts. For example, except for one experiment, all experiments reported by Luria and Delbrück (1943) had a plating efficiency smaller than unity. Their experiment 16 is perhaps the best known by biology students. In that experiment a portion of 0.08 ml was plated from each of the twenty 0.2-ml cultures. Hence, the plating efficiency was ϵ=0.4. The popular genetics textbook of Griffiths *et al.* (2000, p. 481) missed the partial plating feature of that experiment. A recent tutorial (Meneely 2016, p. 373) also mistakenly omitted the partial plating feature of the same experiment. Because 11 of the 20 cultures had no mutants, the tutorial argues by the *P*_0_ method that the mean number of mutations per culture is *m* ∼ 0.6.

Why is the above claim incorrect? There is a crucial distinction between the number of zero mutant counts and the number of actual null cultures—cultures having no mutants prior to plating. Suppose a culture contains one mutant prior to plating. When 40% of that culture is plated, the mutant has a 60% chance of not being counted. Thus, a zero mutant count may result from a nonnull culture. In other words, the experimentalist is uncertain about the number of actual null cultures. To account for this additional source of uncertainty, one can apply a modified *P*_0_ method, which was proposed by Stewart *et al.* (1990, p. 184).

> p0.LD.plating(luria.16.data,e = 0.4)

[1] 0.9786801

Hence, the mean number of mutations per culture is m∼0.98, not 0.60. Luria and Delbrück reported that each culture contained 5.6 × 10^8^ bacteria. Is this the number of cells in the plated portion or in the whole culture? To remove the ambiguity, note that Luria and Delbrück also reported an average mutant count of 28.4 mutants per culture. Because the mean number of the observed mutants in the plated portion was 11.35, the meaning of “per culture” is now clear. There must be 5.6 × 10^8^ cells in the whole culture. That is, *N_t_* = 5.6 × 10^8^. Therefore, the mutation rate is *p* = 0.98/*N_t_* = 1.75 × 10^−9^ mutations per cell division, according to Equation (1).

rSalvador relies on the mutant distribution defined by the PGF $G_{3}(z;m,\epsilon )$ in Equation (5) to compute ML estimates and likelihood ratio C.I.s for the fundamental parameter *m*. It employs the algorithms developed by Zheng (2008). For example, an ML estimate of *m* for Luria and Delbrück’s experiment 16 is

> newton.LD.plating(luria.16.data,e = 0.4)

[1] 1.18636

and an LR-based 95% C.I. for *m* is

> confint.LD.plating(luria.16.data,e = 0.4)

[1] 0.5803079 2.0908012

Many recent investigators were unappreciative toward the effects of partial plating. For example, in the reanalysis of the experiments of Luria and Delbrück (1943), Hamon and Ycart (2012, Table 1) and Ycart (2013, Table 3) ignored the effects of partial plating. In the reanalysis of data from David (1970) and from Werngren and Hoffner (2003), Ycart and Veziris (2014) also disregarded the effects of partial plating. Moreover, unusual care may be required to determine the plating efficiency of an experiment that involves sophisticated procedures such as culture condensation. For instance, the plating efficiency in the study by Werngren and Hoffner (2003) was 0.4, as explained in Zheng (2016a, p. 357). But at first glance the plating efficiency may seem to be 0.2, as was assumed by Mazoyer *et al.* (2017).

Investigators who were aware of the effects of partial plating often relied on an adjustment due to Stewart *et al.* (1990), as this was the only approach recommended on the FALCOR web site. To apply the Stewart approach, the experimentalist first computes an initial estimate m* of *m* by assuming perfect plating efficiency.

> newton.LD(recent.expt)

[1] 15.36162

Because the adjustment factor is A=(ϵ−1)/(ϵlog⁡(ϵ))=21.50, an estimate of *m* is $\hat{m^{*}\;}$× A = 15.36 × 21.5 = 330.24. Note that this estimate of *m* is ∼56% of the corresponding ML estimate (593.6). The reason for this inadequacy of the Stewart approach was given by Zheng (2015b). The above discussion is limited to cases where *w* = 1. Gillet-Markowska *et al.* (2015) proposed applying the Stewart method also to cases where *w* ≠ 1, which is inappropriate in view of the above example. rSalvador currently is unable to deal with such cases.

## Comparing Mutation Rates

Rarely did a researcher perform fluctuation experiments solely to estimate mutation rates. The researcher’s task is often to compare mutation rates across strains or under distinct experimental conditions. Early investigators applied a modified *t*-test to accomplish this task. This practice can lead to misleading *p*-values (Zheng 2015a). Recent investigators turned to the Mann-Whitney test (Ford *et al.* 2013), which also is inappropriate. When applying the Mann-Whitney test to fluctuation assay data, the investigator actually compares the mean numbers of mutations (*m*) between two experiments, not the two mutation rates that need comparing. To compare mutation rates, the investigator must take into consideration the final cell numbers *N_t_*. It is a common feature that *N_t_* values are consistent within each group (*e.g.*, a strain or a particular experimental condition), but they differ noticeably between groups.

rSalvador provides two approaches to mutation rate comparison. The recommended approach is the likelihood ratio test (LRT) for fluctuation assay data, developed by Zheng (2016a). The second is purely empirical; it tests for equality of two mutation rates by checking whether two 84% C.I.s for the mutation rates overlap. If the two 84% C.I.s overlap, the test is not significant at the 5% level. Empirical evidence supporting the latter approach can be found in Zheng (2015a). Consider now the following two experiments from the study of Krašovec *et al.* (2014) (data were kindly provided by R. Krašovec).

expt1 = c(0, 2, 0, 3, 0, 15, 21, 0, 1, 0, 0,

4, 4, 1, 3, 2, 1, 0, 0, 0, 1, 0, 9, 0)

expt2 = c(8, 2, 4, 3, 6, 11, 2, 2, 0, 13, 6, 8)

The investigators measured the relative fitness in both experiments and obtained *w*_1_ = 1.47 and *w*_2_ = 1.45. They also measured the two $N_{t}$ values: *N_t,1_* = 2.27 × 10^8^ and *N_t,2_* = 5.15 × 10^8^. A Mann-Whitney test would yield a *p*-value of 0.01. But this *p*-value is misleading, as cells in the second population underwent about twice as many cell divisions as cells in the first population (the precise ratio is *R* = *N_t_*_,2_ / *N_t_*_,1_ = 2.29). The LRT approach gives a likelihood ratio statistic of 0.19, corresponding to an approximate *p*-value of 0.66.

> LRT.MK(expt1,expt2,w1 = 1.47,w2 = 1.45,R = 2.29)

[1] 0.1924875 0.6608543

Not surprisingly, the 84% C.I.s for the two mutation rates overlap.

> confint.MK(expt1,w = 1.47)/2.27e8

[1] 1.798407e–09 5.244954e–09

> confint.MK(expt2,w = 1.45)/5.15e8

[1] 2.155918e–09 6.105526e–09

Both approaches are applicable to cases where partial plating is adopted. Consider mutant count data from the first (strains H37Rv) and third (strain E 729/94) experiments in the study of Werngren and Hoffner (2003). The plating efficiencies in both experiments were 0.4. The final cell density for the H37Rv strain was 2.3 × 10^8^ cells per ml and that for the E 729/94 strain was 1.3 × 10^8^ cells per ml. Because cultures in both experiments were of the same size (5 ml), the ratio of the two *N_t_* values is therefore *R* = 1.3/2.3. An LRT gives a *p*-value of 0.62.

> LRT.LD.plating(wh.data[[1]], wh.data[[3]], e1 = 0.4,

e2 = 0.4, R = 1.3/2.3)

[1] 0.2435538 0.6216511

Mazoyer *et al.* (2017) proposed a new method based on asymptotic normality of estimated mutation rates. The following simulation study compares statistical power of the three methods. Seven groups of 20-culture fluctuation experiments were simulated using the Lea-Coulson mutant distribution. Each group comprises 10,000 experiments. In the baseline group, *N_t_* = 2 × 10^8^ and the mutation rate is 1.0 × 10^−8^ . In the other groups, *N_t_* = 1 × 10^8^ and the mutation rates are *k* × 10^−8^ for k=1.25,1.5,1.75,2.0,2.5 and 3.0. In all groups, *N*_0_ = 50. Each experiment in the baseline group is compared with the corresponding experiments (*i.e.*, experiments having the same serial number) in all other six groups. Table 1 shows statistical power of the three methods at the 5% significance level. The LRT method and the method of checking overlapping of 84% C.I.s performed almost equally well, but the asymptotic normality method was less powerful.

**Table 1:** Statistical power (Lea-Coulson model)

*p* × 10^8^	1.25	1.5	1.75	2.0	2.5	3.0
LR test	11.0	25.1	45.2	62.8	88.8	97.6
C.I. overlap	10.5	24.7	44.9	62.4	88.7	97.5
Normality	7.93	18.9	35.6	52.8	80.8	93.9

Six groups of simulated experiments were compared with a baseline group. The mutation rate in the baseline group 1.0 × 10^−8^ is smaller than the mutation rates in the six other groups. The final cell population size $N_{t}$ in the baseline group is twice as large as in the other groups. Three comparison methods, namely, the LRT, the method of checking C.I. overlapping, and the asymptotic normality method, were used to test for equality of mutation rates between experiments in the baseline group and experiments from one of the other six groups. Each entry in the table is the percentage of tests that are significant at the 0.05 level. Hence, each entry is an estimate of statistical power at the 0.05 level.

## Accounting for Variability in *N_t_*

There was deep concern that variability in *N_t_* among cultures could interfere with the analysis of fluctuation assay data (Rosche and Foster 2000). However, findings in a recent study (Zheng 2016b) have somewhat lessened worries about that issue. In practice, the experimentalist can use a few cultures to gauge the variability in *N_t_* by calculating the coefficient of variation (CV) for *N_t_*. The new study found that a CV of 0.2 or smaller has a negligible effect on the estimation of mutation rates. More importantly, larger CVs for *N_t_* were rarely encountered in practice. Therefore, if the experimentalist is facing a more critical issue, *e.g.*, partial plating, consideration of variability in *N_t_* should give way to addressing the more critical issue.

Several methods to account for variability in *N_t_* were reviewed in Zheng (2016b). For example, FALCOR refers to a method that requires the experimentalist to measure *N_t_* for all cultures, which often is too laborious to be practical. rSalvador offers a method based on the $B^{0}$ distribution defined by the PGF $G_{4}\left(z;A,k\right)$ in Equation (6). The $B^{0}$ method assumes that the expected number of mutations in a culture is proportional to the *N_t_* of that culture. It further assumes that, conditional on a culture’s *N_t_*, the number of mutants in that culture obeys a Lea-Coulson distribution. If the CV for *N_t_* in an experiment is *C*, then, under additional mild assumptions, the unconditional distribution of the number of mutants is a $B^{0}\left(C^{2}m_{0},C^{\left(-2\right)}\right)$ distribution (Zheng 2016b). Here, *m*_0_ is regarded as the overall mean number of mutations per culture, and one can use rSalvador to make inferences about the parameter *m*_0_. If ${\hat{m}}_{0}\;$is an estimate of *m*_0_, then an estimate of the mutation rate is $\hat{p}=\;{\hat{m}}_{0}\;/{\overset{-}{N}}_{t}$, where ${\overset{-}{N}}_{t}$ is the mean of *N_t_* of all cultures in the experiment.

As an example, suppose that the CV for *N_t_* in the Demerec experiment (Demerec 1945) is 0.15. Point and interval estimates of *m*_0_ are obtained as follows.

> newton.B0(demerec.data,cv = 0.15)

[1] 11.09696

> confint.B0(demerec.data,cv = 0.15)

[1] 8.765365 13.665749

## Determining Sample Size

The question of “How many cultures are needed” must have been asked by investigators countless times, but it has received little attention in the literature. Investigators often choose a sample size on the basis of a published example in which the sample size was determined intuitively. There were no guidelines for determining sample size until recently (Zheng 2017b).

In planning an experiment, the investigator may form a preliminary idea about the magnitude of *m*. A large *m* may reduce the sample size, but it may result in cultures containing more mutants than the investigator is able to count. Zheng (2017b) proposed the following η_500_ index to guide experimental design:

$$\eta_{500}=\text{Prob (a culture having more than 500 mutants)}.$$ (7)

As a rule of thumb, the experimentalist may choose η_500_ < 0.01. Thus, the experimentalist allows no >1% of the cultures to have >500 mutants per culture. As an illustration, assume that the mutation rate to be determined is in the neighborhood of 2 × 10^−7^ mutations per cell division. Assume further that the mutants have a relative fitness of 0.75. If the investigator plans to allow the final number of cells per culture to reach 2 × 10^7^, then *m* would be ∼4.0 according to Equation (1). Therefore, the η_500_ index is

> 1 – sum(prob.MK(m = 4,w = 0.75, n = 500))

[1] 0.001243678

But if the mutants have a relative fitness of 1.2, the η_500_ index will be larger.

> 1 – sum(prob.MK(m = 4,w = 1.2, n = 500))

[1] 0.02221585

If the anticipated value of *m* meets the η_500_ criterion, the experimentalist determines a required sample size by choosing an appropriate ψ score. The ψ score was introduced by Zheng (2017b) as a convenient yardstick for judging the quality of C.I.s. The ψ score is defined by

$$\psi =\frac{\text{half width of a 95\% C.I. for }m}{\text{anticipated magnitude of }m}.\;$$ (8)

Consider a case where w=0.75 has been determined via a fitness assay. The experimentalist further believes that m≈4.0. Choosing a ψ score of 0.25 would lead to an estimated sample size of 31.

> samp.size.MK(m = 4,w = 0.75,psi = 0.25)

[1] 31

If an anticipated value of *m* is large enough to lead to an unacceptable η_500_ index, the experimentalist may resort to partial plating. For example, in the case where m=50 and w=1.0, the η_500_ index is

> 1 – sum(prob.MK(m = 50,w = 1,n = 500))

[1] 0.1894214

However, by plating 10% of each culture, the experimentalist reduces η_500_ to

> 1 – sum(prob.LD.plating(m = 50,e = 0.1,n = 500))

[1] 0.01086645

And the corresponding sample size is

> samp.size.LD.plating(m = 50,e = 0.1)

[1] 15

### Conclusions

Methodological advances made in the past 74 yr have presented the experimentalist with a bewildering array of methods to analyze fluctuation assay data. By providing concrete examples, this article helps the reader make an informed decision about which method is the most appropriate for a given experiment. All discussed methods are approximate, as the underlying mathematical models inevitably depend on simplifying assumptions. Further research will no doubt lead to more flexible methods by relaxing or dropping one or other of these assumptions, *e.g.*, the assumptions (A1)–(A11). However, it is impossible to construct a comprehensive mathematical model by discarding all the 11 assumptions. Even if this herculean feat were accomplished someday, the resultant model would possess these two characteristics: it would still be an approximation to the infinitely complex biological reality, and it would be too cumbersome to be useful in practice. Researchers investigating a challenging biological problem should also play an active part in choosing an appropriate method to analyze their fluctuation assay data, treating the task as an existing, integral part of their research endeavor, not merely as an inescapable, tedious last leg of a long scientific journey.
